# History, chance and selection during phenotypic and genomic experimental evolution: replaying the tape of life at different levels

**DOI:** 10.3389/fgene.2015.00071

**Published:** 2015-02-25

**Authors:** Margarida Matos, Pedro Simões, Marta A. Santos, Sofia G. Seabra, Gonçalo S. Faria, Filipa Vala, Josiane Santos, Inês Fragata

**Affiliations:** Centre for Ecology, Evolution and Environmental Changes, Faculdade de Ciências, Universidade de LisboaLisbon, Portugal

**Keywords:** adaptation, *Drosophila subobscura*, historical constraints, genetic background, genomic convergence, phenotypic convergence

Ever since Darwin, understanding evolutionary processes and patterns have been major scientific quests. In the *Origin of Species*, Darwin explained both adaptation and diversity, and most of his arguments were based on indirect evidence, including comparative approaches. These findings led Darwin to defend that evolution in nature is extremely slow and gradual, hardly being directly observable at the scale of a human generation. Artificial selection, in contrast, was used by Darwin to illustrate the efficacy of natural selection (Darwin, 1859). During the last decades, evolution has been observed in real time. This opened new research possibilities and gave rise to Experimental Evolution, a rapidly expanding field that covers many topics and organisms (Garland and Rose, 2009; Kawecki et al., 2012). The joint power of experimental evolution and recently developed genome-wide tools may now lead us a step further in understanding real-time evolutionary dynamics of populations, both at phenotypic and genomic levels (Baldwin-Brown et al., 2014; Schlötterer et al., 2014). Our contribution to this special issue of *Frontiers in Genetics* focuses on the power of these approaches to assess the role of historical contingencies during adaptation to novel environments, a fundamental subject that has been neglected.

Laboratory experimental evolution studies are powerful because we can follow evolutionary trajectories of independent replicates for one or more traits while controlling for all but the factors under study. For instance this tool is particularly suited to analyze temporal changes using ancestral populations as baselines, generate contrasting phenotypes by divergent selection, and test for predictability of evolution (Kawecki et al., 2012). With such approach, essential questions can be addressed: (1) What is the adaptive potential of populations to novel environments? (2) What is the role of selection and chance during adaptation? (3) What is the tempo and mode of evolution? (4) How constrained is evolution?

With the recent advent of high throughput techniques in genome-wide analysis, also available for non-model organisms (Ellegren, 2014), the field of evolutionary biology is now addressing essential questions more thoroughly: (1) What is the genetic basis of adaptation? (2) Are there many genes of small effect or few genes of major effect involved? (3) What is the role of genetic drift *vs*. selection on candidate genes during local adaptation? (4) What is the mutation rate and how does it change during evolution? (5) Does genomic evolution mimic phenotypic evolution in timing and pattern? (Orr, 2005; Stapley et al., 2010). Until recently, only population genetics modeling and comparative analyses across populations addressed these questions. Both present strengths and limitations (Magalhães and Matos, 2012). The combination of experimental evolution and genomic techniques allows unprecedented resolution to the evolutionary mechanisms underlying phenotypic and genomic change (Burke, 2012; Burke and Long, 2012; Dettman et al., 2012; Lobkovsky and Koonin, 2012; Barrick and Lenski, 2013; Baldwin-Brown et al., 2014; Schlötterer et al., 2014). In particular, these approaches may help us disentangle the role of historical contingencies, the effect of chance events, and the power of selection during adaptation to novel environments.

Selection, history and chance are not mutually exclusive. It is, thus, of utmost importance to define their relative roles in shaping evolution in general, and adaptation to novel environments in particular (Bedhomme et al., 2013). While selection is seen as a deterministic process leading to adaptation, both previous history and chance events are evolutionary contingencies that may lead to disparate, unpredictable results (Lenormand et al., 2009). The classic question of whether evolution is repeatable if we “replay” the tape of life (Gould, 1989) can now be more thoroughly addressed from phenotypes to genomes (Lobkovsky and Koonin, 2012).

The relative role of chance and selection can be tackled by analyzing differences between populations that start from the same ancestral population, while evolving in a novel environment. If selection plays the most important role, it is expected that populations will evolve in parallel, both phenotypically and genotypically, with chance events (e.g., founder effects, genetic drift, and random mutations) having relatively reduced impact.

Experimental evolution studies have shown abundant examples of parallel genomic evolution (Lobkovsky and Koonin, 2012; Stern, 2013), although this is not always the case (Arendt and Reznick, 2008; Elmer and Meyer, 2011). Conspicuous in their abundance are studies of parallel genomic evolution in asexual microorganisms (Tenaillon et al., 2012; Barrick and Lenski, 2013). In experiments starting from the same clone evolution depends on *de novo* mutations (due to a lack of standing genetic variation) and parallelism indicates that adaptation is restricted to a limited number of solutions. It is also possible to follow the fate of lines cryopreserved and see whether evolution repeats itself. Using this approach, Blount et al. (2008) showed the importance of historical contingencies on the evolution of *Escherichia coli*, with the acquisition of a rare key innovation being dependent on prior specific mutations. Studies with sexual organisms are less abundant, but suggest the same tendency for parallel evolution. Such is the case of the genome-wide study of *D. melanogaster* populations selected for accelerated development over 600 generations, with selection repeatedly favoring certain allelic variants (Burke et al., 2010).

While such studies are adequate to test the role of selection and chance events, one important factor that may affect the outcome of adaptation is the evolutionary history of ancestral populations. Indeed, populations with long contrasting histories are expected to present different genetic backgrounds. These genetic differences may affect both their adaptive state and their adaptive potential when a new environment is imposed. Different genetic backgrounds may particularly affect the outcomes of adaptation under rugged landscapes, which is an issue discussed in literature until today (Gavrilets, 2010; De Visser and Krug, 2014). A central question is then: will populations with contrasting histories show convergent evolution, both at the phenotypic and genomic levels, while adapting to novel, common environments?

The distinction between “convergent” and “parallel” evolution is a matter of discussion (see Arendt and Reznick, 2008; Elmer and Meyer, 2011; Stern, 2013). “Convergent evolution” is used here to describe evolutionary patterns and processes by which populations with distinct histories become more similar (at the phenotypic and/or genome-wide levels), when evolving in novel, common environments.

Despite the potential relevance of different histories for convergent evolution during adaptation to novel, common environments, very few studies address this matter. Experiments on phenotypic and genotypic reverse evolution include a study using *Drosophila melanogaster* (Teotónio and Rose, 2000 and Teotónio et al., 2009, respectively), and one using viruses (Bedhomme et al., 2013). Another study addresses convergence at both phenotypic and genome-wide levels using differentiated yeast strains responding to novel environments (Spor et al., 2014). In general, these experiments support the idea that phenotypic convergence is more common than genotypic convergence. Clearly, more empirical work of this kind, particularly using high-throughput sequencing, is required to fully examine the role of historical constraints on evolution and population evolvability.

Previously, we studied laboratory adaptation of *Drosophila subobscura* populations derived from geographically close Portuguese locations (Adraga and Arrábida) throughout several years. We found that all populations adapt during the first generations, though at different rates (Simões et al., 2008a). We have also studied the evolutionary dynamics of several neutral molecular markers in these foundations (Simões et al., 2008b, 2010; Santos et al., 2012, 2013). The joint analyses of molecular markers and life-history traits showed that most differences in adaptive rates were due to sampling effects during the early stages of colonization (Santos et al., 2012). These results illustrate that evolution in a novel environment may be strongly contingent: not only on the initial composition of a newly founded population, but also on the stochastic changes that occur during the first generations of colonization. Nevertheless, in the long run, several life-history traits of experimental populations converged to those of long-established lab populations (Figure 1A). Genomic analysis of these experimental populations will allow us to detail the different contributions of evolutionary forces to convergence.

**Figure 1:**
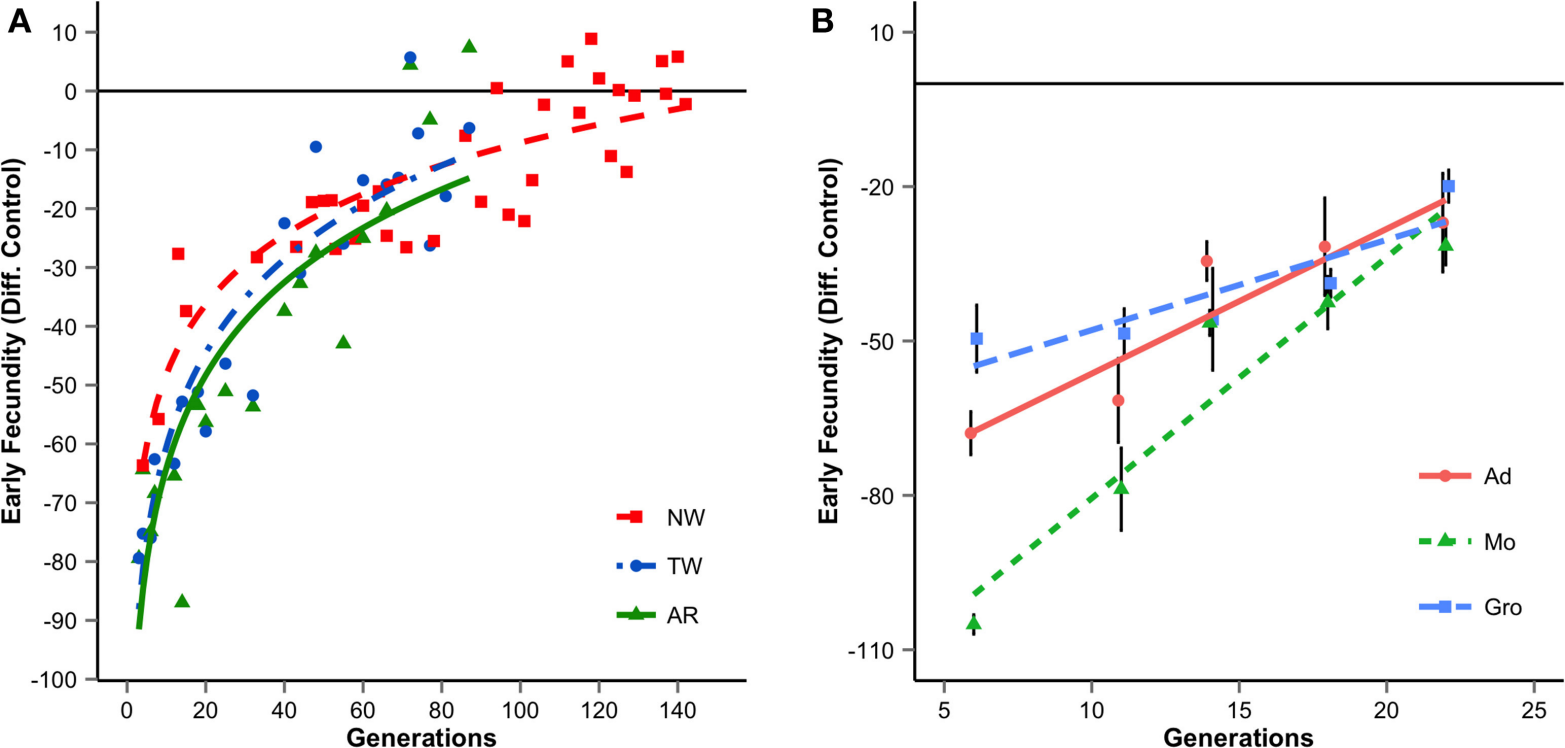
**Evolutionary trajectories of early fecundity (eggs laid during the first week of life) for several foundations since introduction in the laboratory**. The values are the mean differences of the replicate populations per foundation relative to long established laboratory populations. Panel **(A)** results of long-term studies using several foundations of Portuguese populations. Foundations differ in year of collection and/or site (1998: NW, Adraga; 2001: TW, Adraga and AR, Arrábida). Each data point is the average of three replicate populations. **(B)** Short-term studies in populations derived from Groningen (Gro), Montpellier (Mo), and Adraga (Ad) in 2010. Mean values across the three replicate populations of each foundation are presented (adapted from Fragata et al., 2014a). Error bars correspond to differences across replicate populations.

A recent project from our team addressed the impact of history on the dynamics of adaptation (both at phenotypic and genome-wide levels) making use of the fact that *D. subobscura* populations present a clear latitudinal cline in Europe (Rezende et al., 2010). Fly samples were collected from historically differentiated wild populations in three European locations: Groningen (Netherlands), Montpellier (France), and Adraga (Portugal), minimizing sampling effects (cf. Santos et al., 2012) both by increasing the number of founders and by maintaining females (and all their descendants) in separate vials during the first generations. With this set up, the following questions were considered: (1) Will populations that have a different history converge to the same adaptive peak? (2) If so, do they use similar or different molecular routes? (3) Is there a correspondence in tempo and mode of evolution at the phenotypic and genomic levels?

Phenotypic analysis showed that while populations were initially clearly differentiated, they quickly converged during laboratory adaptation in several life-history, physiological, and morphological traits (Fragata et al., 2014a; Figure 1B). As expected from the European chromosomal arrangements cline (Rezende et al., 2010) our populations also presented initial high differentiation in inversion frequencies. Interestingly, and in contrast to what we found for phenotypic traits, history played an important role in the evolutionary dynamics of inversions. Though we obtained clear evidence for the role of selection, after 40 generations of laboratory evolution, populations remained differentiated at the inversion frequencies level (Fragata et al., 2014b). Genome-wide analyses on several generations of these populations are underway to clarify how much convergence occurred at the genome level, and its association with the genetic content of chromosomal inversions. The latter will contribute to a better understanding of the genetic mechanisms underlying the evolution of inversions, a long-term debate in Evolutionary Biology (see Hoffmann and Rieseberg, 2008; Kirkpatrick, 2010).

Increasing the number of studies that couple high-throughput sequencing technologies with experimental evolution is essential. Particularly, designs that involve different long-term histories among populations, as above, will contribute information to a central evolutionary question. How much does history affect adaptation and, ultimately, biological diversity, at both the phenotypic and genomic level? In other words, how predictable is evolution if we replay the tape of life, at different starting points and across biological levels? “(…) *from so simple a beginning endless forms most beautiful and most wonderful have been, and are being, evolved*” wrote Darwin. He would be thrilled to find out in just how much detail we may soon describe the process… – even if ultimately we can't play it back.

## Conflict of interest statement

The authors declare that the research was conducted in the absence of any commercial or financial relationships that could be construed as a potential conflict of interest.
